# Supplementary material for: Long term risk and costs of bleeding in men and women treated with triple antithrombotic therapy–An observational study
Source: PLoS One. 2021 Mar 25;16(3):e0248359. doi: 10.1371/journal.pone.0248359 (PMC7993563; doi:10.1371/journal.pone.0248359)
Supplement: S1 Table — (DOCX) [file pone.0248359.s001.docx]

**Supplementary Table 1. Subgroup analysis of patients with atrial fibrillation**

|  | **All (n=194)** | **Women (n=52)** | **Men (n=142)** | **P-value** |
| --- | --- | --- | --- | --- |
| **Bleeding during follow-up** | | | | |
| Any bleeding | 84 (43.3) | 26 (50.0) | 58 (40.8) | 0.26 |
| **Therapy at first bleeding event** | | | | |
| Triple Antithrombotic Therapy | 37 (44.0) | 9 (34.6) | 28 (48.3) | 0.51 |
| Dual Antiplatelet Therapy | 2 (2.4) | 1 (3.8) | 1 (1.7) |  |
| Aspirin and OAC | 39 (46.4) | 14 (53.8) | 25 (43.1) |  |
| P2Y_12_-inhibitor and OAC | 1 (1.2) | 1 (3.8) | 0 |  |
| Aspirin, single therapy | 1 (1.2) | 0 | 1 (1.7) |  |
| *Figures presented as numbers (percentages). OAC= Oral Anticoagulant.* | | | | |
