# Supplementary material for: Long term risk and costs of bleeding in men and women treated with triple antithrombotic therapy–An observational study
Source: PLoS One. 2021 Mar 25;16(3):e0248359. doi: 10.1371/journal.pone.0248359 (PMC7993563; doi:10.1371/journal.pone.0248359)
Supplement: S2 Table — (DOCX) [file pone.0248359.s002.docx]

**Supplementary Table 2. Bleeding events related to planned TAT duration**

|  |  |  |  |  | p-value |
| --- | --- | --- | --- | --- | --- |
| Planned duration of TAT (months) | 1 | >1 – 3 | >3 - 6 | >6 - 12 |  |
| Bleedings | 65 (38.7) | 34 (45.3) | 10 (37.0) | 0 | 0.49* |
| **p-value bleeders versus non-bleeders* | | | | | |
