# Supplementary material for: Long term risk and costs of bleeding in men and women treated with triple antithrombotic therapy–An observational study
Source: PLoS One. 2021 Mar 25;16(3):e0248359. doi: 10.1371/journal.pone.0248359 (PMC7993563; doi:10.1371/journal.pone.0248359)
Supplement: S3 Table — All co-variates displayed. (DOCX) [file pone.0248359.s003.docx]

**Supplementary Table 3. Cox regression model, time to bleeding. All co-variates displayed.**

|  | **Hazard ratio** | **95% Confidence interval** | **P-value** |
| --- | --- | --- | --- |
| Age (per year) | 1.03 | 1.01 - 1.05 | 0.01 |
| Sex (women vs men) | 1.47 | 0.94 - 2.31 | 0.09 |
| Hemoglobin (per g/L increase) | 0.99 | 0.98 - 1.01 | 0.23 |
| Creatinine (per µmol/L increase) | 1.01 | 1.01 - 1.02 | <0.01 |
| Previous stroke | 0.81 | 0.52 - 1-25 | 0.35 |
| Previous myocardial infarction | 0.98 | 0.65 - 1.48 | 0.94 |
| Warfarin treatment on admission | 0.66 | 0.45 - 0.98 | 0.04 |
