# Supplementary material for: Long term risk and costs of bleeding in men and women treated with triple antithrombotic therapy–An observational study
Source: PLoS One. 2021 Mar 25;16(3):e0248359. doi: 10.1371/journal.pone.0248359 (PMC7993563; doi:10.1371/journal.pone.0248359)
Supplement: Attachment — Submitted filename: Response_to_Reviewers_TAT_PLOSONE_210217.docx [file pone.0248359.s004.docx]

**Respons to reviewers PLOS ONE 17^th^ of Feb 2021**

PONE-D-20-34276
***Long term risk and costs of bleeding in men and women treated with triple antithrombotic therapy – an observational study***

**Reviewer #1**

Comment R1 No 1: Quote: “Most patients (61.8 %) were planned for ≤1 month of TAT followed by 11 months of aspirin and OAC combined, followed by OAC only.” This is a surprising report, as according to the Guidelines when TAT stops OAK and Clopidogrel is recommended combination. This is interesting from the point of treatment strategy in the country of origin of this paper.

Answer R1 No 1: Thank you for this comment. This was actually the guideline recommendation at that time – i.e., to stop either aspirin or P2Y12-inhibition when TAT was stopped. After one year of dual treatment, only OAC continued (this is still the case according to current guidelines).

Comment R1 No 2: Page 5 Baseline characteristics subheading. On admission 60.3% was (were) treated with OAC.

Answer R1 No 2: Thank you, this typo is now corrected.

Comment R1 No 3: Legend for Table 1. Typo: endoxaban should be edoxaban.

Answer R1 No 3: Thank you, this typo is now corrected.

**Reviewer #2**

Comments R2 No 1: Abstract: the authors should state the background of the study in order provide the context of their research.

Answer R1 No 1: Thank you for this comment. The abstract is now updated accordingly.

Comments R2 No 2: Full adjusted cox model of factors associated with bleeding should be reported and displayed.

Answer R2 No 3: Thank you for this comment. The full model is now added as a supplementary file.

Comments R2 No 4: Reductions in bleeding complications have become a primary target for further improvements in both clinical and economic outcomes. Nevertheless, knowledge about the relative size of the costs of bleeding events compared to coronary ischemic, ischemic stroke and/or VTE events in patients treated with TAT is a valuable evidence for decision makers on risk benefits. E.g as shown in table 5 PCI or CABG cost far exceed those of bleeding. Of utmost importance the majority of patients had minor or GI bleeding (e.g. DOACs therapy is more frequently associated with GI bleedings) which are manageable in terms of clinical outcomes as compared with intracranial hemorrhage. The later in addition to an ACS/AMI, ischemic stroke may lead to death or disability which in turn are associated with impaired quality of life and an important chronic and long-term economic burden for both patients/families and health systems. Therefore, the authors should discuss these aspects in the discussion section and limitation in order to give to the readers knowledge about the cost of bleeding relative to those of major events to improve decision making.

Answer R2 No 4: Thank you for this very valuable comment. We have now expanded the discussion, and we think that your input has helped us to strengthen the discussion substantially. We agree that it is a limitation of the study that we do not have complete information on all costs – including indirect costs. We have added this information into the limitation section as well.

Comment R2 No 5: Again, in the discussion section there are too many editor comments. The authors should focus more on discussion their findings.

Answer R2 No 5: Thank you for the comment. We have now changed the discussion section substantially and focused more on our own results.

Comments R2 No 6: The reviewer urges the authors to temper their conclusions. It is not new that TAT is associated with higher bleeding risk and recent guidelines recommend a shorter time of TAT therapy. Nevertheless, the reviewer agrees with the authors that bleeding complications following antithrombotic therapy is associated with higher costs and that there is a need to implement cost-effective strategies in these population

Answer R2 No 6: Thank you for the comment. We have now tempered our conclusions.
